# An advanced and efficient Co_3_O_4_/C nanocomposite for the oxygen evolution reaction in alkaline media†

**DOI:** 10.1039/c9ra07224a

**Published:** 2019-10-23

**Authors:** Abdul Qayoom Mugheri, Aneela Tahira, Umair Aftab, Muhammad Ishaq Abro, Arfana Begum Mallah, Gulam Zuhra Memon, Humaira Khan, Mazhar Ali Abbasi, Imran Ali Halepoto, Saleem Raza Chaudhry, Zafar Hussain Ibupoto

**Affiliations:** Dr M. A. Kazi Institute of Chemistry, University of Sindh Jamshoro 76080 Sindh Pakistan zaffar.ibhupoto@usindh.edu.pk; Department of Science and Technology, Campus Norrkoping, Linkoping University SE-60174 Norrkoping Sweden; Mehran University of Engineering and Technology 7680 Jamshoro Sindh Pakistan; Institute of Physics, University of Sindh Jamshoro 76080 Sindh Pakistan; University of Engineering and Technology Lahore Pakistan

## Abstract

The design of efficient nonprecious catalysts for the hydrogen evolution reaction (HER) or the oxygen evolution reaction (OER) is a necessary, but very challenging task to uplift the water-based economy. In this study, we developed a facile approach to produce porous carbon from the dehydration of sucrose and use it for the preparation of nanocomposites with cobalt oxide (Co_3_O_4_). The nanocomposites were studied by the powder X-ray diffraction and scanning electron microscopy techniques, and they exhibited the cubic phase of cobalt oxide and porous structure of carbon. The nanocomposites showed significant OER activity in alkaline media, and the current densities of 10 and 20 mA cm^−2^ could be obtained at 1.49 and 1.51 V *versus* reversible hydrogen electrode (RHE), respectively. The impedance study confirms favorable OER activity on the surface of the prepared nanocomposites. The nanocomposite is cost-effective and can be capitalized in various energy storage technologies.

## Abstract 2

The design of efficient nonprecious catalysts for the hydrogen evolution reaction (HER) or the oxygen evolution reaction (OER) is a necessary, but very challenging task to uplift the water-based economy.

## Graphical abstract

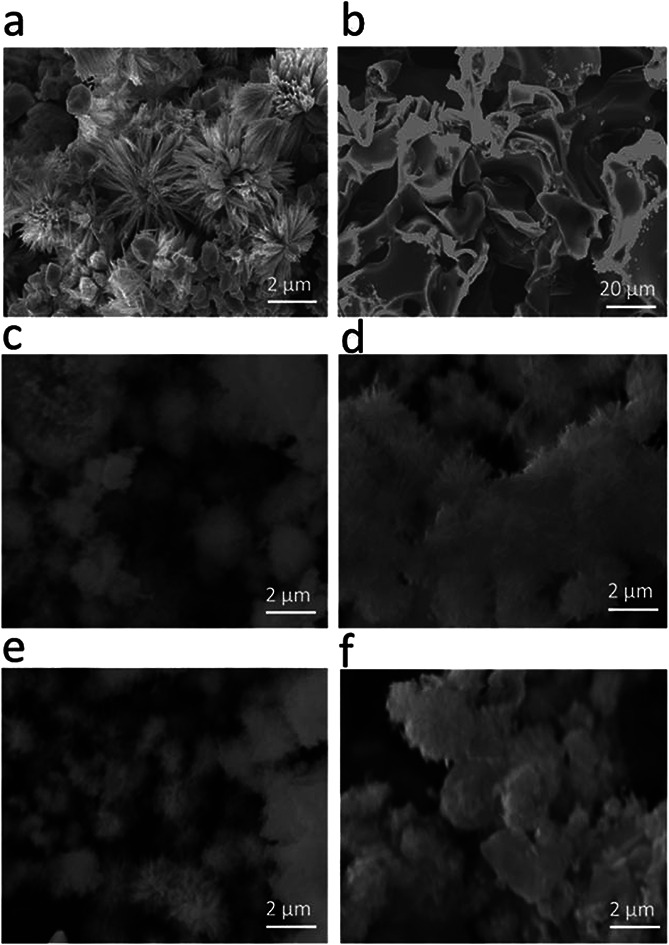

## 1. Introduction

To strengthen the renewable energy reservoirs, electrochemical water splitting is a promising technology and can boost the efficiency of rechargeable metal–air batteries, fuel cells and other energy storage devices.^1–6^ To date, the state-of-art electrocatalysts for the dissociation of water into oxygen are IrO_2_ or RuO_2_.^1,7^ However, the high cost and scarcity of these precious catalysts hinder their application on a large scale. Extensive efforts have been made towards the design of nonprecious, active, and stable OER catalysts using earth-abundant materials, including cobalt phosphate, perovskite oxides and transition metal oxides/layer double hydroxides, for the OER.^8–14^ Moreover, these electrocatalysts are highly stable and exhibit excellent activity in different electrolytes. In the recent past, numerous studies have been reported on the use of earth-abundant metal oxides, including cobalt oxide (Co_3_O_4_),^15^ nickel oxide,^16^ and manganese oxide,^17^ as active materials for the OER. Among these materials, cobalt oxide has been found to be highly efficient and stable.^18^

Co_3_O_4_ nanostructures combined with conducting carbon materials, such as carbon nanotubes, graphene and mesoporous carbon, are widely used as supporting materials due to their high conductivity and high specific surface area.^19–22^ Regardless of this revolutionary advancement, new functional electrocatalysts with low overpotential and high activity are still demanded. The fabrication of carbon nanotubes and graphene is a very expensive and complicated process; therefore, facile approaches are needed to produce carbon materials for the design of efficient composite materials for the production of hydrogen and oxygen gases on a large scale. However, there is no study related to the dehydration of common sugar into carbon and its coupling with cobalt oxide nanostructures. The use of porous carbon materials obtained from the dehydration of sucrose for the coupling of cobalt oxide nanostructures in the design of a functional catalyst is presented in this study. The enhanced performance is mainly attributed to the high density of active edges, which facilitate the OER process at low potential as compared to the pristine carbon and cobalt oxide materials. The improved OER activity is due to the synergetic effect produced between the carbon and cobalt oxide nanostructures due to the quantum confinement effect.

Herein, we present a simple strategy for the design of a new functional nanocomposite based on the combination of cobalt oxide nanostructures with porous carbon materials. The nanocomposite was successfully characterized by various analytical techniques such as X-ray diffraction, Fourier transform infrared spectroscopy, scanning electron microscopy and energy dispersive spectroscopy. The presented nanocomposites showed excellent OER activity at 1.49 V *vs.* RHE to achieve the current density of 10 mA cm^−2^ in a 1 M KOH electrolyte. The impedance study revealed fast OER kinetics over the proposed nanocomposites.

## 2. Experimental

Cobalt nitrate hexahydrate, urea, sulfuric acid, sucrose sugar, potassium hydroxide and 99.5% ethanol were used without any further purification. The synthesis of cobalt oxide and carbon composite was carried out in three steps. Step 1: the carbon material was obtained by the dehydration of sucrose in the presence of an 8 M concentrated sulfuric acid solution. The carbon material was washed several times with deionized water followed by ethanol treatment. The carbon material was dried overnight at 60 °C. Step 2: the deposition of cobalt hydroxide nanostructures on porous carbon was carried out by a hydrothermal method. An equimolar solution of cobalt nitrate hexahydrate and urea was prepared in 100 mL of deionized water in four separate beakers. Then, successive addition of porous carbon with different weights, such as 100 mg, 200 mg, 300 mg, and 400 mg, which were labeled as samples 1, 2, 3, and 4, respectively, was carried out. The precursor solution was tightly covered with an aluminum foil and kept in an electric oven at 95 °C for 5 hours. After the completion of growth, the cobalt hydroxide nanostructures were obtained by filtration and washed with deionized water. The samples were dried at room temperature. Step 3: the calcination of all the samples was carried out at 500 °C for three hours, and then, these samples were ready for application in the catalysis process. In a similar way, the pristine cobalt oxide nanostructures were synthesized without the addition of carbon materials.

The morphology of the as-prepared nanostructured materials was investigated by the JEOL scanning electron microscope at the accelerating voltage of 20 kV. The crystalline and phase purity was explored by the Philips powder diffractogram at room temperature. Fourier transform IR technique was used to confirm the interaction between cobalt oxide and porous carbon material. Energy dispersive spectroscopy was used to monitor the elemental composition of composite catalysts. The electrochemical experiments were performed in a 1 M KOH aqueous solution. The catalyst ink was prepared by mixing 15 mg of the prepared composite in 2.5 mL of deionized water and 1 mL of glutaraldehyde as a binder polymer. The geometric area of the glassy carbon electrode was 3 mm. The drop cast method was used to modify the surface of the glassy carbon electrode using 10 μliter of each catalyst ink. The modified electrode was dried in a preheated oven at 60 °C. The platinum wire and silver–silver chloride (Ag/AgCl) saturated with the KCl electrolyte were used as the counter and reference electrodes, respectively. The cyclic and linear sweep voltammetry were used as the primary modes for the investigation of the OER activity. Electrochemical impedance spectroscopy was carried out in the frequency range from 1000 kHz to 0.1 Hz at the amplitude of 10 mV and onset potentials of 0.5 V in a 1 M KOH electrolyte.

All the potentials reported throughout the manuscript were converted to RHE through the Nernst equation as follows:

1 *E*_RHE_ = *E*_Ag/AgCl_ + 0.059pH + *E*^0^_Ag/AgCl_.

The current density was calculated by dividing the current with the geometric area of the glassy carbon electrode.

## 3. Results and discussion

### 3.1. Structural characterization of the as-obtained Co_3_O_4_-based electrocatalysts

**Fig. 1:**
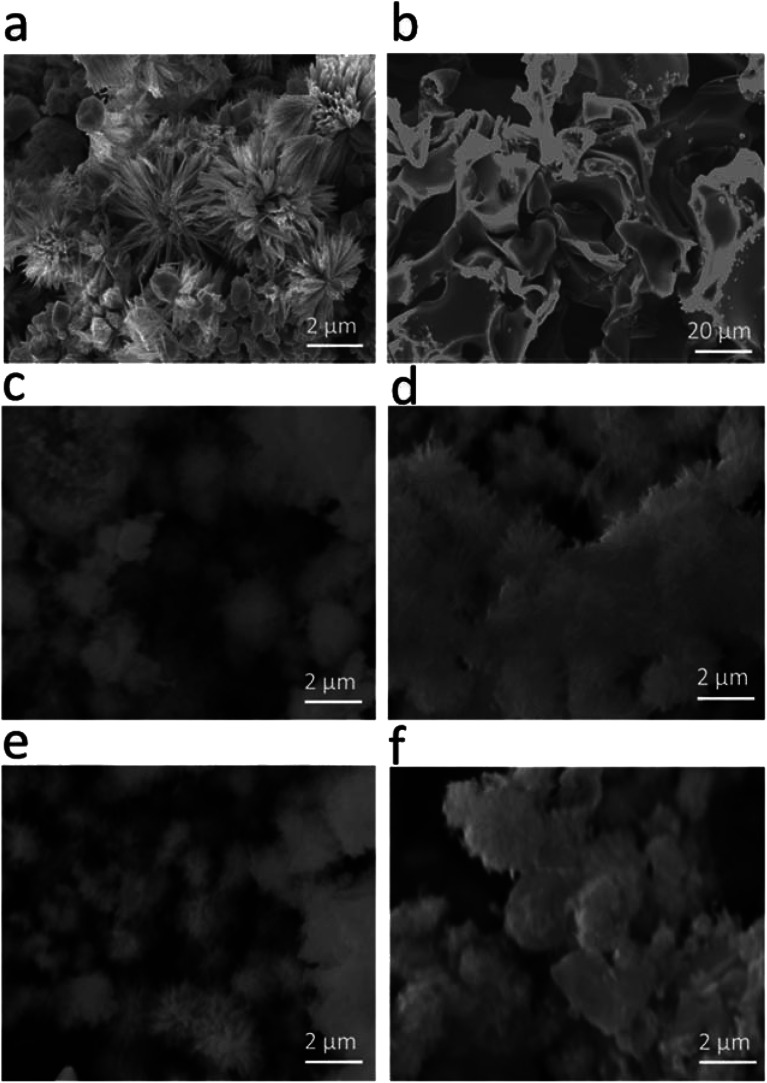
SEM images: (a) pristine cobalt oxide, (b) pure carbon, (c) sample 1, (d) sample 2, (e) sample 3, and (f) sample 4.

**Fig. 2:**
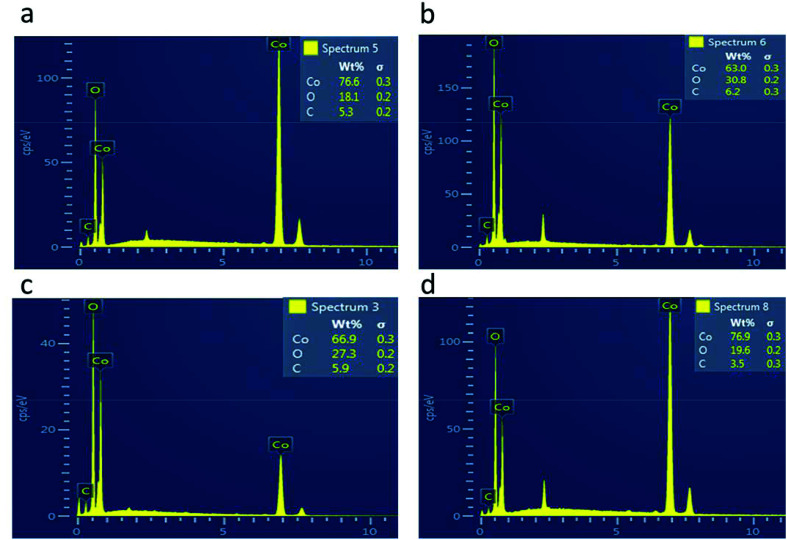
EDS spectra: (a) sample 1, (b) sample 2, (c) sample 3, and (d) sample 4.

The morphology of the as-prepared samples was investigated by scanning electron microscopy, as shown in Fig. 1. Fig. 1(a) shows the morphology of the pristine cobalt oxide sample that exhibits the morphology of a bundle of nanowires. The morphology of the carbon material before the deposition of cobalt oxide is shown in Fig. 1(b), which is a porous structure. The sharp edges are found for the cobalt oxide nanostructures on porous carbon, as depicted in Fig. 1(c–f). The variation in the carbon content did not alter the morphology of the cobalt oxide nanostructures. The Co_3_O_4_/C composite structure showed a large number of active sites for catalyzing the water splitting reaction. The chemical composition of the as-prepared Co_3_O_4_/C composite materials was analyzed by energy dispersive spectroscopy, and the results indicated the presence of Co, O and C as the main elements, as shown in Fig. 2. No other impurities were found, and the EDS results were consistent with those of the XRD analysis.

**Fig. 3:**
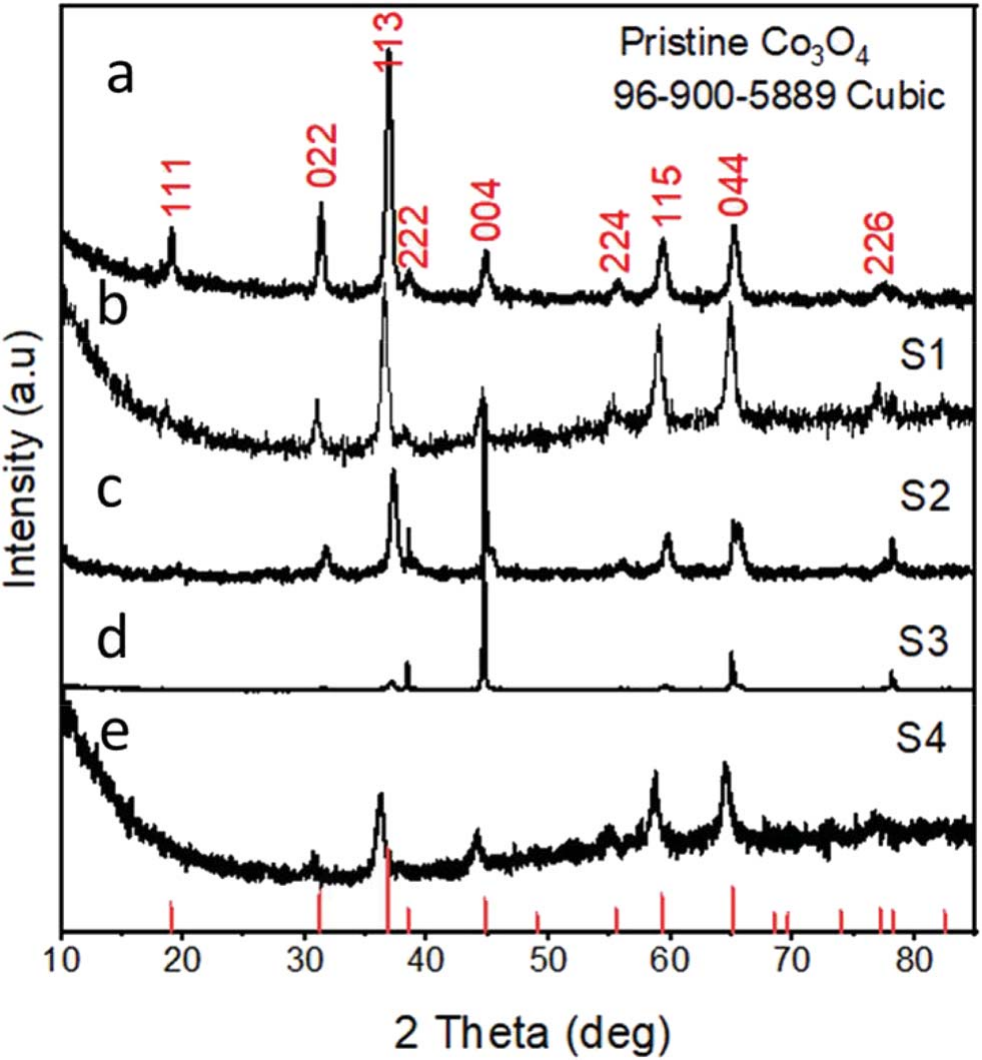
XRD spectra: (a) sample 1, (b) sample 2, (c) sample 3, (d) sample 4, and (e) pristine Co_3_O_4_.

Fig. 3 shows the XRD diffraction patterns of the as-prepared Co_3_O_4_ samples. Fig. 3(a) shows the reflection peaks for pristine Co_3_O_4_, and this confirms the cubic phase of Co_3_O_4_. All the diffraction patterns corresponded to those of Co_3_O_4_ and fully agreed with the standard JCPDS card no: 96-900-5889. The XRD study was also carried out for the various Co_3_O_4_/C composite samples, which also confirmed the cubic phase of cobalt oxide, as shown in Fig. 3(b–e). All the diffraction patterns are also in good agreement with the standard JCPDS card no: 96-900-8589. No other phases or impurities were detected by XRD. The XRD patterns for the pure carbon material are shown in S1,† and the sample exhibits an amorphous phase.

**Fig. 4:**
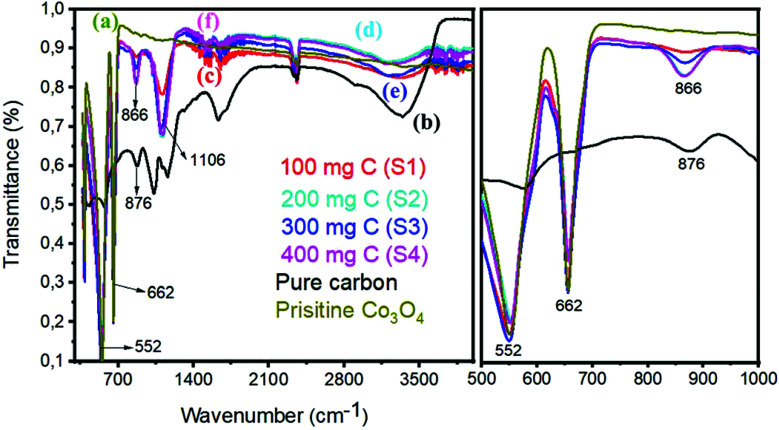
FTIR spectra: (a) pristine cobalt oxide, (b) pure carbon, (c) sample 1, (d) sample 2, (e) sample 3, and (f) sample 4. Right hand part indicates the zoom in region from 500–1000 cm^−1^.

Fig. 4 shows the FTIR spectrum of pristine Co_3_O_4_ and Co_3_O_4_/C composite samples. The right part of Fig. 4 indicates the magnified region from 500 to 1000 cm^−1^ of the FTIR spectrum. The FTIR study shows that the spectrum of the Co_3_O_4_/C composite does not exhibit any new absorption peak, and this validates that the structure of cobalt oxide and carbon is not damaged. The sharp peaks at 552 cm^−1^ and 662 cm^−1^ of pristine cobalt oxide are assigned to the Co–O stretching vibration modes. Similar stretching vibrations are noticed in the composite samples, confirming the strong interaction between Co_3_O_4_ and carbon material. The bands at 1649 cm^−1^ of pristine Co_3_O_4_ are indexed to the vibration of C=C. The composite samples also have C=C of Co_3_O_4_/C and a large difference, suggesting the electronic interaction between C=C and Co–O. The absorption bands in the region of 1000–1500 cm^−1^ are due to the O– symmetric and asymmetric stretching and the C–O stretching vibrations, and these features indicate that the adsorption of H_2_O and CO_2_ is unavoidable as the sample preparation was conducted in open air. In the case of pure carbon, the C–C, C=C, C–O, and C–H stretching vibrations are prominent. We believe that the bonding features between Co_3_O_4_ and carbon material are due to the *in situ* growth of cobalt oxide.

### 3.2. OER activity in alkaline media

**Fig. 5:**
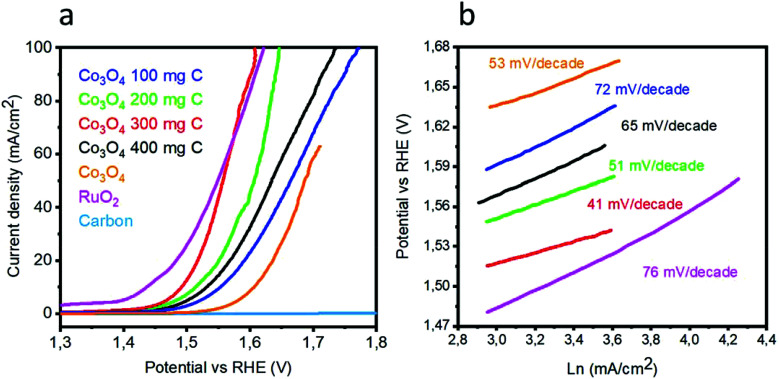
(a) Linear sweep voltammetry of pristine cobalt oxide, pure carbon, sample 1, sample 2, sample 3, and sample 4, RuO_2_ at the scan rate 5 mV s^−1^ in 1 M KOH, (b) corresponding Tafel plots.

To investigate the OER activity of various catalysts based on the Co_3_O_4_/C composite and pristine Co_3_O_4_ in a 1 M KOH electrolyte, LSV polarization curves were obtained at the scan rate of 1 mV s^−1^, as shown in Fig. 5(a). Pristine cobalt oxide showed a slow OER activity at higher onset potential, but the addition of carbon as a supporting material for the deposition of Co_3_O_4_ nanostructures not only reduced the onset potential but also resulted in higher current density. The lowest overpotential to achieve a current density of 10 mA cm^−2^ is 1.49 V *vs.* RHE for the sample 3. The excellent OER activity of the composite catalysts can be attributed to the high surface area with improved active edges in the presence of the porous carbon material. In addition to the sample 3, the sample 1, 2, and 4 are efficient catalysts for the OER but require slightly higher OER potential. The OER activity is improved with the addition of carbon material. However, after a certain amount, the carbon material facilitates the aggregation of cobalt oxide nanostructures; this decreases the OER performance of the composite sample, as shown for the sample 4. It is believed that in the wet chemistry process, a nanostructured material has more defects, and further, the combination of these defect materials with a porous material can lead to enhanced OER performance, as demonstrated by the sample 3. It can be observed from Fig. 5(a) that peroxidation peaks are assigned to M^2+^ (M–OH; herein, M is = Co/Ru) and M^3+^ (M–OOH) oxidation, which can be found in the range from 1.25 to 1.43 V *vs.* RHE. It is believed that the height of the pre-oxidation peak current or the integrated peak area provides the density of the active edges (M–OOH) available within the metal oxide, and consequently, a favorable OER performance is achievable. The composition of each catalyst is solely responsible for the position of the pre-oxidation peak; thus, it varies from sample to sample.^23–25^ It is very difficult to know all the information about the OER mechanism, and generally, the OER process on transition metal oxides in alkaline media has been proposed by Li *et al.*^26^ and is expressed in following chemical equations:

2 M* + OH^−^ → M*OH + e^−^

3 M*OH + OH^−^ → M*O + H_2_O + e^−^

4 M*O + OH^−^ → M*OOH + e^−^

5 M*OOH + OH^−^ → M*O_2_ + H_2_O + e^−^

6 M*O_2_ → M* + O_2_

**Fig. 6:**
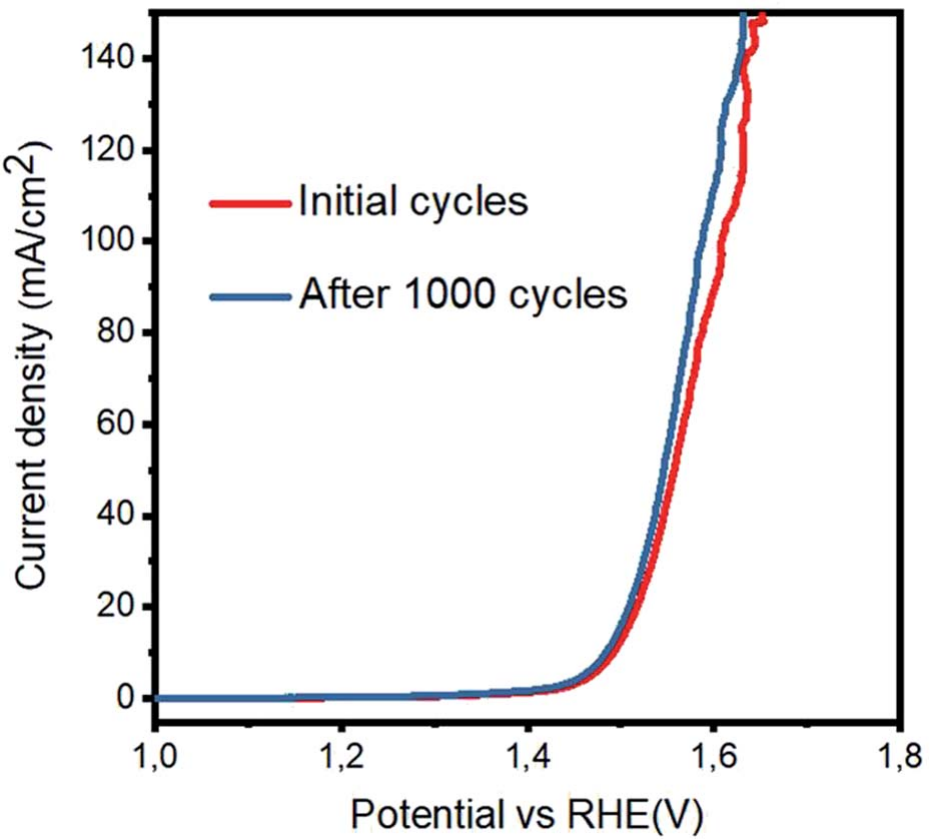
Durability experiment *via* cyclic voltammetry at the scan rate of 20 mV s^−1^ after 2000 cycles in 1 M KOH.

Herein, M* indicates the active edges on cobalt oxide, and the step (4) is considered as the rate-determining step; moreover, the Volmer, Heyrovsky and Tafel reactions correspond to the Tafel slope values of 120, 40 and 30 mV dec^−1^, respectively.^27,28^ The sample 3 has a lower Tafel slope value of 41 mV dec^−1^ than RuO_2_, which suggests that the OER kinetics has a more favorable environment on the sample 3 and it follows the Volmer–Heyrovsky reaction mechanism, as shown in Fig. 5(b). Based on the polarization curves, it can be said that only metal oxide is responsible for the OER kinetics, and porous carbon only enhances the performance of cobalt oxide by providing more surface for the growth of cobalt oxide nanocrystals, which may be involved in the adsorption of OH^−^ on the surface and its further conversion into oxygen gas. In addition to this, the Tafel slopes of RuO_2_, the samples 1, 2, and 4 and pristine Co_3_O_4_ were found to be 76, 51, 72, 65, and 53 mV dec^−1^, respectively. Based on the available literature and to the best of our knowledge, the OER results are encouraging and ensure the possible configuration of the electrolyzer for efficient water splitting devices. These observations of superior OER results are mainly attributed to the high surface area provided by the carbon material during the deposition of the Co_3_O_4_ nanostructures. It is safe to say that the composite materials may have enhanced conductivity, synergetic effect produced from the active sites of Co_3_O_4_ and more surface area of the carbon material for the favorable OER activity. The durability and stability of the sample 3 was investigated through cyclic voltammetry for the OER, as shown in Fig. 6. After 1000 CV runs at 100 mV s^−1^, no significant loss in the catalytic activity was found, but a negligible loss of potential was visible at higher current densities.

**Fig. 7:**
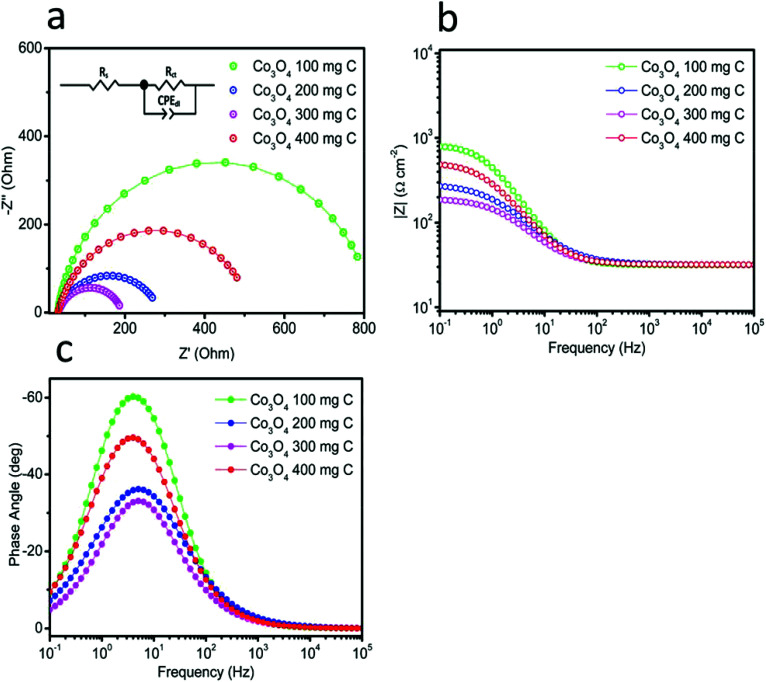
Electrochemical impedance spectroscopy in the frequency range from 100 kHz to 0.1 Hz in 1 M KOH at the amplitude of 10 mV: (a) Nyquist plots, inset showing the fitted equivalent circuit model, and (b and c) Bode plots.

Catalytic activity is strongly associated with the charge transfer resistance. Therefore, electrochemical impedance spectroscopy was carried out at high, intermediate and low frequencies to understand the kinetics of charge transfer and the recombination mechanisms at the interfaces of the electrode and electrolyte. Fig. 7(a) shows the Nyquist plots obtained for the catalysts in the frequency range from 0.1 Hz to 1 MHz under an applied potential of 1.45 V *vs.* RHE. The same data is also plotted in the Bode format, as shown in Fig. 7(b) and (c). This impedance plots can be explained with the help of an equivalent circuit model, as depicted in inset of Fig. 7(a). The circuit fitting values are provided in Table 1. *R*_s_ corresponds to the solution resistance, which is nearly similar for all the electrocatalysts. The diameter of the semicircle corresponds to the charge transfer resistance (*R*_ct_). The quick charge transfer process and faster kinetics of the reaction are due to the lower *R*_ct_ values. The obtained *R*_ct_ values for Co_3_O_4_ 100 mg C, Co_3_O_4_ 200 mg C, Co_3_O_4_ 300 mg C and Co_3_O_4_ 400 mg C are 792, 258, 162 and 486 Ω, respectively. The result shows that Co_3_O_4_ 300 mg C corresponds to a faster reaction rate than the other electrocatalysts. Single peak is observed in the Bode plots with the corresponding relaxation being assigned to the charge transfer process. The maximum oscillation frequency (*f*_max_) of the impedance semicircle of Co_3_O_4_ 300 mg C is less than that of the other electrocatalysts, thus the corresponding electron recombination lifetime (*τ*_n_ = 1/2π*f*_max_) increased Co_3_O_4_ 300 mg C can be attributed to favorable adsorption on the working electrode.^29–31^ The EIS observations revealed favorable OER activity on the composite samples. The performance of the produced catalyst was compared with that of the reported efficient catalysts, as presented in Table 2, and the produced catalyst was superior in terms of the Tafel slope and overpotential.

**Table 1:** The fitted circuit values

	*R*_s_ Ω	*R*_ct_ Ω	CPE_dl_ mF
100	31.56	792.30	0.30
200	31.82	258.20	0.54
300	31.78	162.00	0.83
400	31.88	486.30	0.45

**Table 2:** Comparison of the presented overall water splitting results of the electrocatalyst (sample 3) with those of the recently reported studies

Catalyst	Overpotential mV	Tafel slope mV dec^−1^	Reference
CoCo LDH	393	59	*Nat. Commun.*, 2014, **5**, 4477
CoO_*x*_@CN	∼385		*J. Am. Chem. Soc.*, 2015, **137**, 2688
Co–P films	345	47	*Angew. Chem. Int. Ed.*, 2015, **54**, 6251
Co_3_O_4_/NrmGO	310	67	*Nat. Mater.*, 2011, **10**, 780
Ni_*x*_Co_3−*x*_O	∼370	59–64	*Adv. Mater.*, 2010, **22**, 1926
N-G-CoO	340	71	*Energy Environ. Sci.*, 2014, **7**, 609
Co_3_O_4_/C	260	41	This work

## 4. Conclusions

In summary, herein, simple Co_3_O_4_/C composite-based catalysts were designed by the dehydration of common sucrose followed by the wet chemical method for the *in situ* growth of the Co_3_O_4_ nanostructures. The composite material has shown excellent OER activity, which can be assigned to high surface area, large number of active centers, synergetic effect and rich defect chemistry within the catalyst structure. The simple integration of interface between Co_3_O_4_ and the carbon material could facilitate the flow of water molecules for dissociation and consequently support the adsorption of intermediate species. Particularly, the OER could be conducted at low overpotentials by achieving the 10 mA cm^−2^ current density at 1.49 V *vs.* RHE. The lowest possible Tafel slopes are achieved for the nonprecious catalysts, which highly indicate the capitalization of composite materials for practical applications. The proposed strategy of designing the catalysts is very simple and cost-effective and can be capitalized on a large scale to produce oxygen.

## Conflicts of interest

Authors declare no conflict of interest in this research work.

## Supplementary Material

RA-009-C9RA07224A-s001
