# Supplementary material for: An advanced and efficient Co3O4/C nanocomposite for the oxygen evolution reaction in alkaline media
Source: RSC Adv. 2019 Oct 23;9(59):34136–43. doi: 10.1039/c9ra07224a (PMC9073646; doi:10.1039/c9ra07224a)
Supplement: RA-009-C9RA07224A-s001 [file RA-009-C9RA07224A-s001.pdf]

Electronic Supplementary Material (ESI) for RSC Advances.
This journal is © The Royal Society of Chemistry 2019

Advanced and efficient Co_3O_4/C nanocomposite for the oxygen evolution reaction in alkaline media

Abdul Qayoom Mugheri[a], Aneela Tahira[b], Umair Aftab[c], Muhammad Ishaq Abro[c], Arfana Begum Mallah[a], Gulam Zuhra Memon[a], Humaira Khan[a], Mazhar Ali Abbasi[d], Imran Ali Halepoto[d], Saleem Raza Chaudhry[e], Zafar Hussain Ibupoto*[a]

[a] Dr. M.A Kazi Institute of Chemistry University of Sindh Jamshoro, 76080, Sindh Pakistan

[b]Department of Science and Technology, Campus Norrkoping, Linkoping University, SE-60174 Norrkoping, Sweden

[c]Mehran University of Engineering and Technology, 7680 Jamshoro, Sindh Pakistan

[d]Institute of Physics, University of Sindh Jaamshoro, 76080, Sindh Pakistan

[e]University of Engineering and Technology, Lahore

*Corresponding author: Zafar Hussain Ibupoto

Email address: zaffar.ibhupoto@usindh.edu.pk

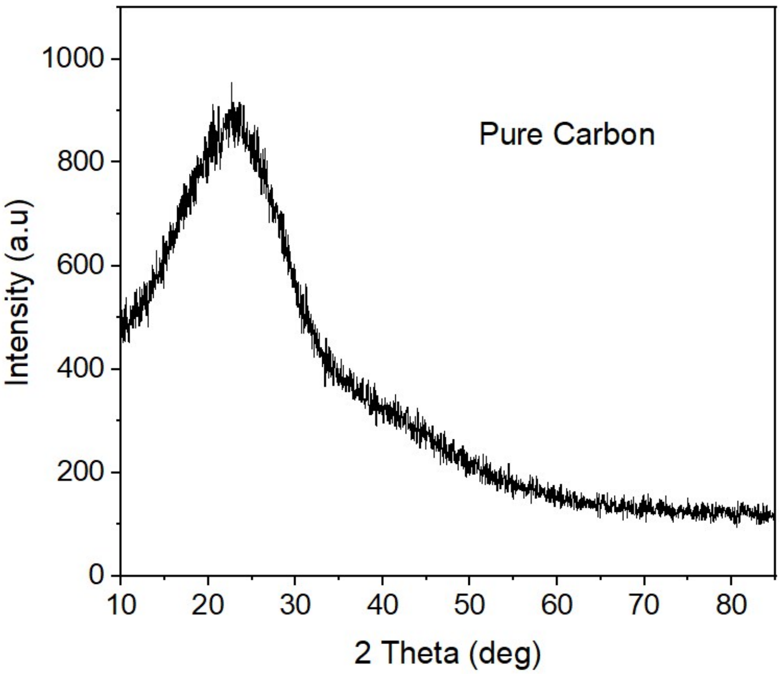

S1: XRD patterns of pure carbon obtained from the dehydration of sucrose
